# The Ministry's Suggestions

**Published:** 1920-01-24

**Authors:** 


### The Ministry's Suggestions.

Attention is drawn by the Town Clerk of Southwark to a recent circular of the Ministry of Health, pointing out that the establishment of the system of medical inspection of school children has disclosed the fact that a large number of children, on first admission to school, were found to be suffering from preventable ailments and defects which were likely to retard their development, and were in a large degree attributable to insufficient knowledge which the infant welfare centre was specially designed to meet. The Ministry, therefore, considered that the centres should provide for the systematic supervision of the health of babies and little children until they are old enough to attend school and come automatically under the care of the school medical officer.

The Ministry expressed its readiness to make a grant in aid of, and look to the council to provide (*a*) adequate lying-in accommodation; (*b*) hospital treatment for infants; (*c*) provision of accommodation in convalescent homes for women recovering from confinement and for some conditions in young children; and (*d*) provision of homes, etc., for attending to the health of children under five years of age, of widowed, deserted, and unmarried mothers.

The effect is to make the infant welfare centre responsible for looking after babies and little children until they are over five years of age, when, on admission to school, they automatically come under the care of the school medical officer.

